# Characterization of Dextran Biosynthesized by Glucansucrase from *Leuconostoc pseudomesenteroides* and Their Potential Biotechnological Applications

**DOI:** 10.3390/antiox12020275

**Published:** 2023-01-26

**Authors:** Renpeng Du, Liansheng Yu, Meng Sun, Guangbin Ye, Yi Yang, Bosen Zhou, Zhigang Qian, Hongzhi Ling, Jingping Ge

**Affiliations:** 1 Engineering Research Center of Agricultural Microbiology Technology, Ministry of Education & Heilongjiang Provincial Key Laboratory of Plant Genetic Engineering and Biological Fermentation Engineering for Cold Region & Key Laboratory of Microbiology, College of Heilongjiang Province & School of Life Sciences, Heilongjiang University, Harbin 150080, China; 2 State Key Laboratory of Microbial Metabolism, School of Life Sciences and Biotechnology, Shanghai Jiao Tong University, Shanghai 200240, China; 3 School of Basic Medical Sciences, Youjiang Medical University for Nationalities, Baise 533000, China

**Keywords:** glucansucrase, dextran, *Leuconostoc pseudomesenteroides*, in vitro, characterization

## Abstract

Glucansucrase was purified from *Leuconostoc pseudomesenteroides*. The glucansucrase exhibited maximum activity at pH 5.5 and 30 °C. Ca^2+^ significantly promoted enzyme activity. An exopolysaccharide (EPS) was synthesized by this glucansucrase in vitro and purified. The molecular weight of the EPS was 3.083 × 10^6^ Da. Fourier transform infrared (FT-IR) and nuclear magnetic resonance (NMR) spectroscopy showed that the main structure of glucan was 97.3% α-(1→6)-linked D-glucopyranose units, and α-(1→3) branched chain accounted for 2.7%. Scanning electron microscopy (SEM) observation of dextran showed that its surface was smooth and flaky. Atomic force microscopy (AFM) of dextran revealed a chain-like microstructure with many irregular protuberances in aqueous solution. The results showed that dextran had good thermal stability, water holding capacity, water solubility and emulsifying ability (EA), as well as good antioxidant activity; thus it has broad prospects for development in the fields of food, biomedicine, and medicine.

## 1. Introduction

Polysaccharides, being renewable functional biomolecules, are widely used in the food, medicine and cosmetics industries as well as in other fields because of their roles in antioxidant activity, immune regulation, probiotics and the prevention of environmental pollution [1,2,3]. Plants, animals and microorganisms are all major sources of polysaccharides [4]. Polysaccharides from different sources not only differ significantly in yield but also have unique structural and functional properties. Microorganisms, especially lactic acid bacteria (LAB), have the advantages of ease of cultivation, high polysaccharide production and strong probiotic effects that have widely attracted attention from researchers [5]. At present, the main methods for obtaining polysaccharides include direct extraction from animals and plants and microbial fermentation [6]. These methods are complex and inefficient. In recent years, the rapid development of synthetic biology technology has given rise to new opportunities for the synthesis of polysaccharides and research on their structure–activity relationships [5,7]. The enzymatic synthesis of polysaccharides in vitro can significantly increase their yield [8].

Due to the complex metabolic system of LAB, the synthesis of polysaccharides is jointly regulated by a variety of genes and proteins. Glucosyltransferases (GTF, EC 2.4.1.5) isolated from *Leuconostoc*, *Lactobacillus* and *Streptococcus* species transfer D-glucopyranosyl units with sucrose as donor molecules under appropriate conditions [9,10]. *Leuconostoc mesenteroides* glucosyltransferase (commonly called glucansucrase) is used to produce glucan polymers and oligosaccharides and is widely used in a variety of industrial fields [11]. Glucansucrase appears to be a sucrose-induced extracellular enzyme [12]. Thus, glucan synthesis can be carried out in vitro without the need for energetic cofactors, using only purified glucansucrase and sucrose. This complex can be used to efficiently prepare dextran with a controlled molecular weight (Mw) [13]. Therefore, the establishment of a stable and efficient technology for dextran biosynthesis can not only increase dextran yield but also promote the study of the relationship between its structure and function.

In a previous study, a dextran was produced by *Leuconostoc pseudomesenteroides* DRP-5 and the dextran is a highly linear dextran with α-(1→6) linkages [14], but the biosynthesis mechanism of dextran was not elucidated. Glucansucrase is the key enzyme in dextran biosynthesis. There have been many reports on glucansucrase. However, studies have found that glucansucrase from different strains have great differences in structure and properties. The catalytic and biochemical properties of glucansucrase have not been thoroughly studied. Therefore, exploring the relationship between glucansucrase and exopolysaccharide (EPS) is an important premise to analyze the mechanism of EPS biosynthesis. Moreover, some studies have shown that glucansucrase easily forms complexes with polysaccharides which are not easy to separate, which brings great difficulties in separation and purification. The molecular weight of the glucansucrase is relatively large and it is difficult to use molecular biological methods to clone pure enzyme. In this paper, the enzymatic properties of cell-associated glucansucrase from the *Ln. pseudomesenteroides* strain DRP-5 and the kinetic parameters of its bio-catalysis are discussed. Additionally, a method of enzymatic synthesis of dextran in vitro is established and the differences in structure and properties between natural dextran and dextran synthesized using glucansucrase with sucrose as the carbon source are analysed.

## 2. Materials and Methods

### 2.1. Bacterial Cultivation

*Ln. pseudomesenteroides* DRP-5 was stored in 30% glycerol at −80 °C. The strain was isolated in MRS liquid medium by the three-sector streak method and cultured after 18 h.

### 2.2. Purification of Glucansucrase

As previously described, *Ln. pseudomesenteroides* was cultured in 1 L MRS-S fermentation medium at 30 °C for 48 h [14]. Cell-free supernatant (CFS) containing crude glucansucrase from the bacterial strain was obtained by centrifugation at 8000× *g* for 15 min at 4 °C. Crude glucansucrase was separated and purified by ammonium sulfate precipitation, dialysis and ion exchange chromatography (DEAE-cellulose FF, S8800, Sigma, St. Louis, MI, USA) and gel filtration (Sephadex G-75, S8186, Sigma, St. Louis, MI, USA). The purified glucansucrase fraction was concentrated using ultrafiltration centrifuge tubes and subsequently characterized further. The SDS-PAGE was used to determine the molecular weight. Glucansucrase activity was determined in 50 mM pH 5.4 sodium acetate buffer including 1 mM CaCl_2_ and 100 mM sucrose at 37 °C [15]. One glucansucrase unit (U) was defined as the amount of enzyme capable of releasing 1 mmol/min of fructose from sucrose.

### 2.3. Effect of Temperature, pH and Metal Ions on the Activity of Purified Glucansucrase

The optimal temperature for the purified glucansucrase was determined by incubating the enriched glucansucrase in 20 mM phosphate buffer (pH 6.5) at temperatures ranging from 20 °C to 55 °C for 30 min and then assaying the enzyme activity. At 4 °C, the enzyme activity was 100% and other samples were taken as reference. Glucansucrase was tested under standard test conditions of pH 4–8.5. Briefly, 1 mL of the enriched purified glucansucrase was mixed with 1 mL buffer of varying pH. The glucosucrase-buffer mixture was incubated at 30 °C for 30 min, and the activity of the enzyme was measured. The effect of metal ions (Na^+^, K^+^, Mg^2+^, Zn^2+^, Ca^2+^, Mn^2+^, Cu^2+^, Ba^2+^, Hg^2+^ and Fe^3+^) on glucansucrase activity was also tested. For this, 1 mL of 2 mM metal ion solutions were mixed with 1 mL purified glucansucrase and incubated for 30 min at 30 °C, following which the enzyme activity was determined. The enzyme activity of a sample in 20 mM sodium acetate buffer (pH 5.5) at 30 °C was given a value of 100%, and the activity of the sample mixed with various metal ions was compared to this control value.

### 2.4. Synthesis of EPS In Vitro

EPS was synthesized in vitro by incubating 2 mL of 5 U/mL purified glucansucrase in 100 mL of 20 mM sodium acetate buffer (pH 5.5) containing 2 mM CaCl_2_ and 3 g sucrose at 30 °C for 24 h. The reaction solution was placed in an 80 °C water bath and incubated for 30 min to inactivate unreacted glucansucrase. The proteins were precipitated with equal volumes of 10% (*w*/*v*) trichloroacetic acid (TCA) followed by centrifugation at 10,000× *g* for 30 min at 4 °C. The supernatant and three volumes of cold 95% (*w*/*v*) ethanol was mixed and placed at 4 °C for 24 h, followed by centrifugation at 10,000× *g* for 30 min at 4 °C. The EPS pellet was redissolved in milliQ water and subjected to dialysis through a membrane with a porosity of 8000–14,000 Da against milliQ water for 2 days at 4 °C and then lyophilized. A UV-vis spectrometer (UV-3300, MAPADA, Shanghai, China) was used to detect the purity of EPS.

### 2.5. Determination of Chemical Composition

The phenol-sulfuric acid method was used to determine the content of total carbohydrates in EPS with glucose as the benchmark [16]. The Bradford method determined the protein content [17]. The sulfuric acid-carbazole method [18] and barium chloride-gelatin method were used to determine the [19] content of uronic acid and sulphate group proportions, respectively. The carbon, nitrogen, hydrogen and sulfur content were determined using an elemental analyzer (Vario EL Cube, Elementar, Ronkonkoma, NY, USA).

### 2.6. Molecular Mass Distribution

The average molecular number (Mn) of the purified EPS was detected by gel permeation chromatography (GPC; 1515, Waters, Milford, MA, USA) combined with refractive index detection. The stationary phase of this method is porous gel, the mobile phase is 0.1 M NaNO_3_, detection was performed using a differential multiangle laser light scattering instrument (DAWN EOS, Wyatt, Shanghai, China) and the detectors were RI and MALS. The column temperature was 45 °C, Ohpak SB-804 HQ analytical columns (F6429103, Ohpak, Shanghai, China) were used and the loading volume was 100 μL. Data were collected and processed using the GPC/SEC software (TDAmaxViscotec). The Mw was calculated according to the standard glucose calibration curve.

### 2.7. Monosaccharide Composition Analysis

Two milligrams of purified EPS powder were hydrolyzed at 120 °C for 1 h with 1 mL of 2 M trifluoroacetic acid (TFA), following which the residual acid was removed by three rounds of methanol vacuum distillation. The composition of the hydrolysate was analyzed by HPLC (LC-20AT, Shimadzu, Japan), with acetonitrile and water used as the mobile phase in a 75:25 ratio. The separation was carried out at 24 °C with a flow rate of 0.8 mL/min and sample volume of 20 µL. Monosaccharides such as glucose, arabinose and rhamnose were used as standards.

### 2.8. Fourier Transform Infrared (FT-IR) Spectroscopy Analysis

Purified EPS powder and KBr were mixed at a ratio of 1:100 and then ground, placed in a mold and pressed into a transparent sheet using a hydraulic press. FT-IR spectra were recorded with a Nicolette iS10 spectrometer in the wavelength range of 400–4000 cm^−1^.

### 2.9. X-ray Diffraction (XRD) Analysis

To determine the crystallinity of the EPS, XRD was employed at varying 2θ angles (10–80°), then 50 mg sample of EPS was placed on a quartz support and peak intensity measurements were performed at room temperature.

### 2.10. Nuclear Magnetic Resonance (NMR) Spectroscopy Analysis

Thirty milligrams of purified EPS disappeared in 0.5 mL of 99.96% D_2_O. ^1^H NMR, ^13^C NMR and HSQC spectra were performed using a Bruker AVANCE 400 MHz spectrometer (Bruker, Billerica, MA, USA) operated at 25 °C with a 5 mm inverse probe.

### 2.11. Scanning Electron Microscopy (SEM) and Atomic Force Micrograph (AFM) Analysis

SEM images of the purified dextran sample were obtained with S-4800 SEM equipment (Hitachi, Japan). The dextran powder was conductively bonded, sputtered with gold and measured with a tungsten source starting at 5 kV and with an accelerating voltage. The image magnifications were 200× and 1000×. Then, 10 mg of purified dextran was made to disappear in MilliQ water (1 mL) and stirred repeatedly to obtain a uniform dispersion. About 50 μL dextran solution was dispersed on a mica sheet and N2-dried to remove the water at room temperature. Subsequently, AFM images were obtained using a scanning probe microscope (Bruker, Berlin, Germany) in tapping mode.

### 2.12. Thermal Analysis

The thermal properties of dextran were investigated using a Maia F3 200 device (Netzsch, Selb, Germany). Then 50 mg dried dextran samples were loaded into an Al_2_O_3_ crucible and sealed, following which the crucible was heated at a rate of 10 °C/min from 40 °C to 800 °C in a dynamic inert nitrogen atmosphere of 50 mL/min.

### 2.13. Water Solubility Index (WSI) and Water Holding Capacity (WHC) Analysis

The WSI of dextran was determined using the assay method of Wang et al. [20,21] with slight modifications—45 mg of dextran was made to disappear in 0.5 mL of MilliQ water and shaken with a vortex shaker for 2 h to obtain a homogeneous suspension. Insoluble dextran was obtained by centrifugation at 10,000× *g* for 30 min at 4 °C, which was then freeze-dried and weighed. Determination of the *WHC* of dextran was as previously described: 30 mg of dried dextran was made to disappear in 0.5 mL of MilliQ water and the samples were incubated at 30 °C for 30 min to allow for uniform dispersion. Centrifuging at 10,000× *g* for 30 min at 4 °C took place, then blot drying of the insoluble glucose and wiping with filter paper. The initial weight was represented by *M*_1_ and the precipitate after freeze-drying by *M*_2_.

$$WHC(\%)=\frac{M1-M2}{M2}\times 100$$

### 2.14. Emulsification Activity (EA) Analysis

The EA assay was performed as described by Zhao et al. [22]. Briefly, 2.5 mL of 1 mg/mL purified dextran solution and 2.5 mL hydrocarbons or oils (diesel, gasoline, hexane, benzene, soybean oil and sunflower oil) were mixed. The mixtures were stirred to form homogeneous solutions and left to stand for 48 h at 4 °C. The emulsion heights were measured at 24 h and 48 h.

### 2.15. Antioxidant Activity Analysis

Using Vc as a proportional ratio, the scavenging effect of dextran on DPPH radical, hydroxyl (•OH) radical, O^2−^ radical and ABTS was detected by the method of Jiang et al. [23] and Pei et al. [24] in the concentration range 0.5–6 mg/mL.

## 3. Results

### 3.1. Purification and Characterisation of Glucansucrase

**Figure 1:**
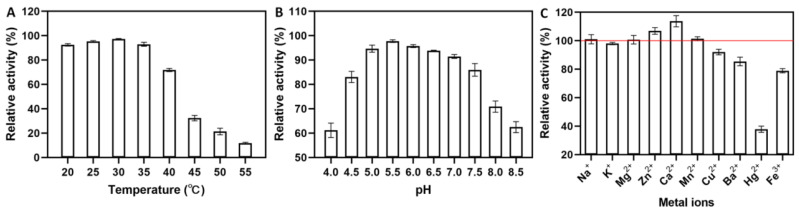
Enzymatic properties of *Ln. pseudomesenteroides* glucansucrase. (**A**) Temperature stability; (**B**) pH value stability; (**C**) metal ions stability.

The glucansucrase was eluted as a single symmetrical peak. The results showed that the specific activity of sucrase was 313.86 U/mg and the purification rate was 14.59 times. The SDS-PAGE analysis showed that the glucansucrase formed a single band with Mw 170 kDa, indicating that it had high purity. The Mw of DRP2-19 glucansucrase was consistent with the previous literature (Mw is between 150 and 220 kDa) [25]. The effect of temperature on glucansucrase activity is shown in Figure 1A. The purified glucansucrase from *Ln. pseudomesenteroides* exhibited optimum activity at 30 °C. This result is consistent with the previously reported optimal reaction temperatures of glucansucrase from *Lactobacillus plantarum* DM5 [26] and *Pediococcus pentosaceus* SPA [27], both at 30 °C. However, a different observation was recorded by Song et al. [28] and López-Munguía et al. [29]. They found optimal temperatures of 45 °C and 40 °C for glucansucrase from *Ln. citreum* SK24.002 and *Ln. mesenteroides* NRRL B-1355, respectively, both higher than our result. In this study, there was a loss of activity on either side of 30 °C. These results indicated that the thermal stability of the pure enzyme decreased sharply over 40 °C. Low temperatures had little effect on purified glucansucrase activity and the glucansucrase was stable between 4 °C and 35 °C.

The effect of pH on purified glucansucrase was also investigated (Figure 1B). The maximum relative activity of purified glucansucrase was observed at pH 5.5. A rapid decrease in glucansucrase activity was observed at pH values above 7.5. The optimum pH for the activity of glucansucrase from *Ln. citreum* SK24.002 [28] and recombinant *Ln. mesenteroides* TDS2-19 [30] was around 5.5, consistent with the results of this study. The above results revealed that the purified glucansucrase from *Ln. pseudomesenteroides* had higher activity at lower pH compared to other glucansucrases from *Ln. dextranicum* NRRL B-1146 [31] and *P. pentosaceus* SPA [27].

The effects of different metal ions on the activity of purified glucansucrase showed that Ca^2+^ and Zn^2+^ improved enzyme activity. Of these, Ca^2+^ significantly increased glucansucrase activity by about 113% (Figure 1C). A similar study reported that Ca^2+^ can activate glucansucrase, yielding a relative activity of 123.83 ± 5.11% [30]. Na^+^, Mg^2+^ and Mn^2+^ ions did not significantly influence glucansucrase activity. The glucansucrase activity was slightly inhibited by K^+^ and markedly inhibited by Cu^2+^, Ba^2+^, Hg^+^ and Fe^3+^, which is in accordance with observations by Wu et al. [18] and Das et al. [26]. The above results suggest that some metal ions have activating and inhibitory effects on glucansucrase.

### 3.2. Analysis of the EPS Produced by Ln. pseudomesenteroides Glucansucrase

**Figure 2:**
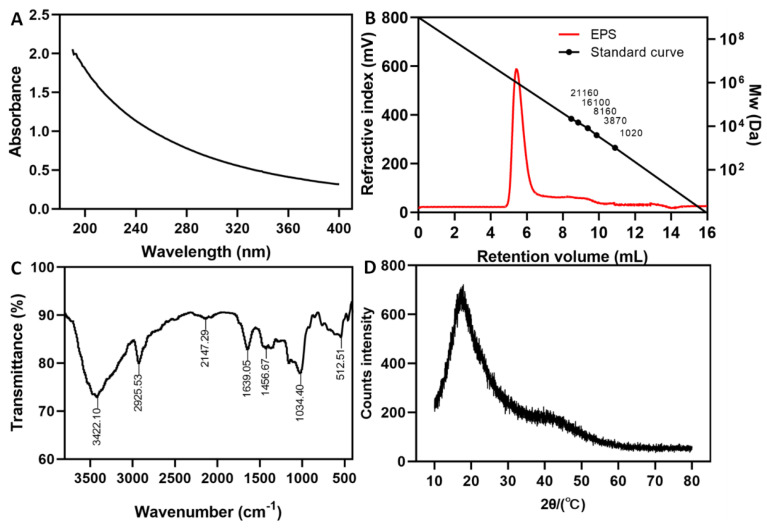
The purity and structural characteristics of dextran. (**A**) UV-vis spectrum; (**B**) GPC chromatogram; (**C**) Fourier transform infrared (FT-IR) spectrum; (**D**) X-ray diffraction (XRD) spectrum.

The purified glucansucrase produced EPS with sucrose in vitro. The EPS formed a white and fluffy solid after lyophilization and was soluble in milliQ water, giving rise to clear or slightly opalescent solutions of considerable viscosity. The sucrose utilization rate was about 96%. There was no absorption observed in the UV-vis spectrum of the EPS solution, indicating that there was no nucleic acid or protein in the EPS (Figure 2A). The carbohydrate content of the EPS was 100%, consistent with the corresponding value for the *Weissella confusa* XG-3 EPS [32] and higher than that for the EPS from *Leu. citreum* B-2 [33] and *Ln. mesenteroides* DRP105 [34]. The carbon, oxygen, nitrogen and hydrogen contents were 49.19%, 43.06%, 1.27% and 5.84%, respectively. This result is in line with previous reports on *Ln. citreum* B-2 EPS [33] and *Ln. lactis* L2 EPS [22]. The EPSs isolated from *Ln. citreum* B-2 and *Ln. lactis* L2 contained a small amount of sulfur. This was because the EPS was obtained by strain fermentation, isolation and purification, which was greatly affected by external factors such as medium composition, strain characteristics and the method used for isolation and purification. However, because this study only used glucansucrase to synthesize the EPS in vitro, the process was not affected by the external environment and technical methods, leading to a higher purity.

### 3.3. Molecular Mass and Monosaccharide Composition Analysis of EPS

A single symmetrical peak appeared on the GPC elution curve, indicating that the purified EPS was homogeneous. The Mw and Mn of the EPS were 3.083 × 10^6^ Da and 2.199 × 10^6^ Da and its EPS is similar to that of *Ln. pseudomesenteroides* XG5 (2.6 × 10^6^ Da) [35] and higher than *L. plantarum* JLK0142 (1.34 × 10^5^ Da) EPS [36]. However, the values were significantly lower than those of the EPS from *Ln. pseudomesenteroides* DRP-5 (6.25 × 10^6^ Da) [14], *Ln. mesenteroides* TDS2-19 (8.79 × 10^7^ Da) [37] and *Ln. lactis* L2 (3.70 × 10^6^ Da) [22]. Some studies have shown that the sucrose concentration, synthesis method and synthesis conditions can affect the molecular weight of EPSs. In addition, enzymatic biosynthesis of EPS and natural EPS may be different and regulated by a combination of enzymes. An EPS with a low molecular mass has the advantages of good water solubility and strong biological activity, while an EPS with a high molecular mass has the characteristics of high viscosity and stability. Through the analysis of the monosaccharide composition of EPS, it was found that the structure of EPS and other *Leuconostoc* glucans are similar [22,35,37]. The EPS of *Leuconostoc* is dominated by glucan and there are few studies on other species.

### 3.4. FT-IR and XRD Analysis of EPS

The functional groups of the EPS were analyzed by FT-IR spectroscopy. The FT-IR spectrum of purified EPS is shown in Figure 2C. The very broad band at 3422.10 cm^−1^ shows the presence of O-H groups. A band at 2925.53 cm^−1^ corresponded to the antisymmetric stretching vibrations of C-H [38]. Absorption bands at 1639.05 cm^−1^ and 1456.67 cm^−1^ were assigned to the C=O and C-O stretching vibrations of a COO group, respectively [39]. Bands between 1200–1000 cm^−1^ are distributed on the carbohydrate ring [40]. The absorption band of pyranose ring in EPS polymer is 1034.40 cm^−1^ [41]. A band at 512.51 cm^−1^ indicated the existence of glycosidic linkage bonds. The result was very similar to the natural EPS from *Ln. pseudomesenteroides* DRP-5 [14].

The XRD analysis of the purified EPS is shown in Figure 2D. A major broad peak emerged near 20° (2θ) in the XRD curve, suggesting the non-crystalline amorphous nature of the EPS, which is in agreement with observations by Zhao et al. [32]. Mathivanan et al. [42] also found, based on polar peaks in the XRD curve, that the *Bacillus cereus* KMS3-1 EPS was amorphous.

### 3.5. NMR Analysis of EPS

**Figure 3:**
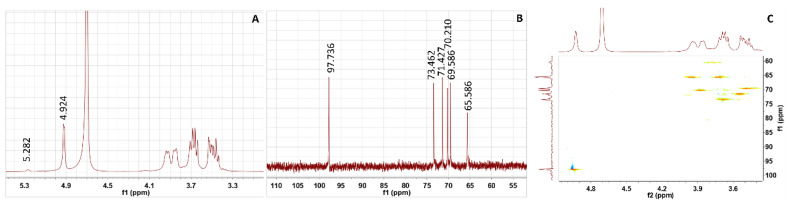
Nuclear magnetic resonance (NMR) spectrum of dextran. (**A**) ^1^H spectrum; (**B**) ^13^C spectrum; (**C**) HSQC spectrum.

The NMR spectra of the purified EPS are shown in Figure 3. In the ^1^H NMR spectrum, an anomeric signal associated with typical glucosyl residues was detected at 4.924 ppm, confirming the presence of α-(1→6) glucosyl residues [43,44]. Additionally, a low-intensity anomeric signal of δ 5.282 ppm was assigned to α-(1→3). The results showed that the relative intensities (%) of the two peaks were 97.3% (δ 4.924 ppm) and 2.7% (δ 5.282 ppm). A strong signal at 4.64 ppm in the spectrum originated from D_2_O (Figure 3A) [45]. In the ^13^C NMR spectrum, six signal peaks at 97.736 ppm, 73.462 ppm, 71.427 ppm, 70.210 ppm, 69.586 ppm and 65.586 ppm were assigned to the C-1, C-3, C-2, C-5, C-4 and C-6 of the EPS, respectively (Figure 3B) [46].

HSQC can detect protons of 4.92/97.73 (H1/C1), 3.55/71.42 (H2/C2), 3.67/73.46 (H3/C3), 3.43/69.58 (H4/C4), 3.86/70.21 (H5/C5) and 3.95, 3.70/65.58 (H6, H6′/C6), and the C-correlation peak directly connected to it indicates that there are α-(1→6) glycosyl residues in the repeat unit of EPS (Figure 3C). This study confirmed that EPS synthesis from *Ln. pseudomesenteroides* glucansucrase is a glucose-based glucan with 97.3% α-(1→6)-linked D-glucopyranoid in its backbone and with 2.7% α-(1→3) branching. The structure of the EPS was similar to that produced by *Ln. mesenteroides* NTM048, which was constituted of an α-(1→6)-linked glucose with α-(1→3) branches [47]. However, the EPS from *Ln. pseudomesenteroides* DRP-5 is a highly linear dextran with α-(1→6) glycosidic bonds [14]. Kang et al. [48] found that the dextransucrase from *Ln. mesenteroides* B-1299CB4 synthesized an α-(1→6) glucan containing branches of α-(1→3) and α-(1→4) glucosidic linkages. Bejar et al. [49] reported that the EPS synthesized by glucansucrase from *Weissella* sp. TN610 was a linear dextran made of α-(1→6). Kralj et al. [50] found that glucansucrase from *L. reuteri* 121 synthesized a unique, highly branched glucan with α-(1→6) and α-(1→4) glucosidic bonds. The results showed that glucansucrases from different strains can synthesize EPSs with different structures. Moreover, the process of dextran biosynthesis may be regulated by various enzymes, which accounts for the structure difference between glucansucrase-synthesized dextran and natural dextran from *Ln. pseudomesenteroides* DRP-5.

### 3.6. SEM and AFM Analysis of Dextran

**Figure 4:**
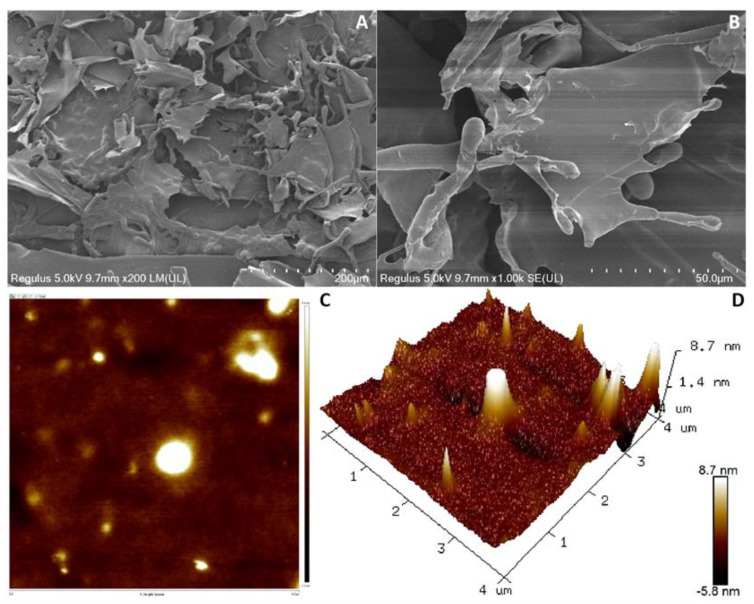
SEM and AFM showing the surface morphology and morphological features of dextran. (**A**) ×400 magnification of SEM; (**B**) ×2000 magnification of SEM; (**C**) planar view of AFM; (**D**) cubic view of AFM.

The surface and microstructures of the dextran were characterized by the SEM technique. As can be seen from Figure 4A,B, the microscopic images of the dextran microstructure show a dense, smooth and irregular flake, but the natural DRP-5 dextran presented a highly branched, hollow tubular and smooth glittering surface structure [14]. Many tubular branches were found on the surface of the dextran chains at higher magnification. Smoothed dextran increases the WHC and viscosity of food [51]. The sheet and branch microstructure allows the dextran to have a higher water capacity and is expected to be crucial to its mechanical properties and biological activity in the form of texturizers, thickeners, viscosifiers and stabilizing agents utilized in the food and pharmaceutical industries. A similar structure was also found in the natural EPS from *L. plantarum* WLPL04 [52].

AFM can also be applied to the analysis of the surface roughness and three-dimensional structure of EPS, which provides a basis for its physical and morphological properties. An AFM image of dextran in water is given in Figure 4C,D, which shows that the surface of dextran is relatively dense and has several peaks with heights ranging from 2 nm to 8.7 nm. The production of such protrusions is mainly caused by dextran macromolecules in molecules or between molecules, which have strong affinity for water molecules and have a certain pseudoplasticity [39]. EPS from *Streptococcus thermophilus* CC30 has a similar structure [53]. This property illustrates the potential of dextran in the production of water-retaining films and biodegradable plastic films.

### 3.7. Thermal Analysis of Dextran

**Figure 5:**
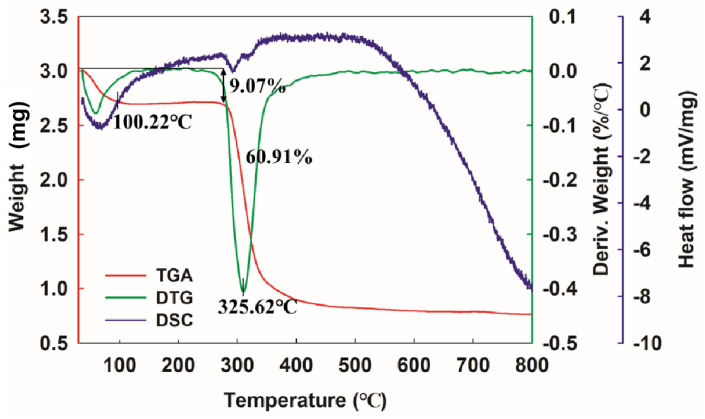
The thermal properties of dextran. Red curve is TGA, green curve is DTG, purple curve is DSC.

The thermal properties of EPS are a key factor in its industrial application. Figure 5 shows the results for the thermal properties of dextran. In the TGA, the purified dextran showed a three-step degradation trend. In the first step, weight loss (9.07%) was observed between 40 °C and 100 °C, which was attributed to the loss of water. In the second step, dramatic weight loss (60.91%) occurred from 280 °C to 400 °C, which might be because of the depolymerization of the EPS.

DTG analysis showed that the thermal decomposition reaction temperature (Td) of glucan was 325.62 °C, higher than that of the natural DRP-5 dextran (298.1 °C) [14], *L. fermentum* S1 EPS (316.9 °C) [54] and *W. confusa* PP29 dextran (305 °C) [55] which could be attributed to differences in molecular mass, monosaccharide composition and molecular structure. As the temperature increased further, weight was no longer lost. This phenomenon is related to the complexity of the EPS molecular structure. The DSC curve showed that a significant melting absorption peak appears at the early stage of heating at 100.22 °C, which was caused by (endothermic) evaporation of water [56]. This is consistent with the TGA and DTG curves. The high Td of dextran indicates that it is ideal for application in the food industry.

### 3.8. WSI and WHC Analysis of Dextran

The WSI value of dextran was 94.08 ± 1.52% and the WHC was 402.71 ± 3.84%, which were higher than the corresponding values for the EPS of *Ln. lactis* KC117496 [57] and *Lactococcus lactis* F-mou [58]. Moreover, the WSI and WHC of the dextran were different from that of natural DRP-5 dextran [14]. The water-soluble dextran had a good WHC, which was attributed to its absorptive structure, which holds a lot of water and is hydrogen-bonded. A previous study found that the WHC of yoghurt was greatly improved when the yoghurt starter was co-cultured with EPS-producing bacteria [59]. These results show that dextran has physical properties such as viscosity, water solubility and other forms of solubility that make it helpful in improving the structure and rheology of food [57].

### 3.9. EA Analysis of Dextran

EA is an essential characteristic for maintaining the stability of two immiscible substances. The EA of dextran on hydrocarbons and oils is shown in Table 1. The results showed that dextran had good EA against the tested hydrocarbons and oils. The change in EA over time was significant. In this study, 83.81 ± 0.56% EA was observed for soybean oil, which is higher than the EA of the EPS from *L. helveticus* MB2-1 [38] and *Ln. citreum* B-2 [33], which showed values of 36.2 ± 0.2% and 22.05 ± 0.47%, respectively. Dextran’s high stability and good EA indicate that it may be used as an emulsifier in the food and petroleum industries. In addition, the high hydrocarbon EA of glucan can improve the removal of hydrocarbons from the ecosystem [60].

### 3.10. Antioxidant Activity Analysis of Dextran

**Figure 6:**
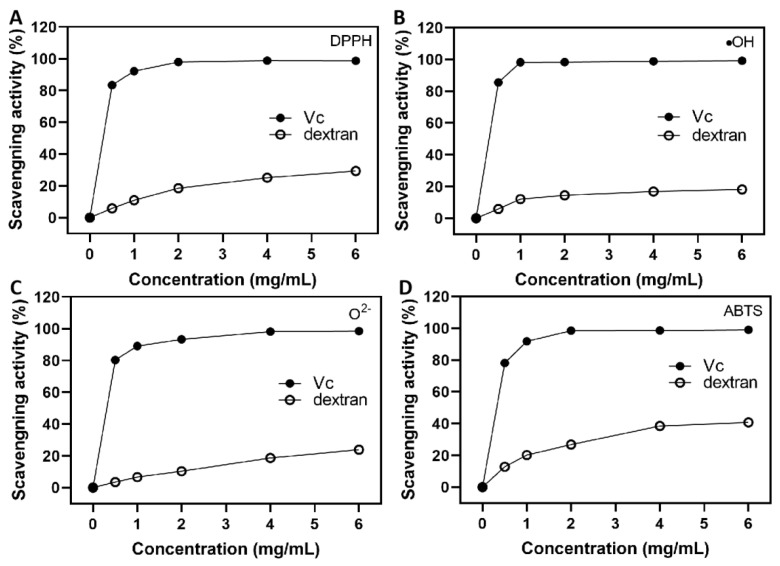
The antioxidant activity of dextran. (**A**) Scavenging ability of DPPH; (**B**) scavenging ability of •OH; (**C**) scavenging ability of O^2-^; (**D**) scavenging ability of ABTS.

The antioxidant activity of dextran is shown in Figure 6. The results showed that the scavenging ability of DPPH radical, •OH radical, O^2-^ radical and ABTS^+^ was significantly enhanced by dextran. At 6 mg/mL, the scavenging effects were 29.56 ± 1.82%, 18.13 ± 1.04%, 23.85 ± 2.77% and 40.46 ± 1.61%, respectively, lower than that of the EPS from *Ln. lactis* L2 [23]. The scavenging activity of the positive control group Vc was always higher than that of the dextran. The scavenging activity was over 98% when the concentration was 2 mg/mL. Wang et al. [33] found that the *Ln. citreum* B-2 EPS also had antioxidant properties and reported similar results to our study. However, this dextran showed good ABTS scavenging activity (Figure 6D). ABTS is a stable free radical that can reflect the body’s antioxidant capacity. At a concentration of 4.0 mg/mL, the removal rate was 38.65 ± 1.72%, consistent with the test results of the EPS from *Ln. pseudomesenteroides* DRP-5 [14]. Dextran exhibited different levels of antioxidant activity that could be attributed to its chemical composition, functional groups, molecular mass and chain conformation and branching structures. Previous studies have shown that the lower the Mw, the greater the antioxidant capacity of the EPS [54]. Therefore, this study concludes that the high Mw of dextran is an important factor influencing its antioxidant capacity.

## 4. Conclusions

In this study, a glucansucrase was purified from *Ln. pseudomesenteroides*. Maximum glucansucrase activity was found at pH 5.5 and 30 °C and enhanced by Ca^2+^ ions. Dextran was synthesized in vitro by glucansucrase using sucrose as the raw material, with a structure comprising 97.3% α-(1→6) bound D-glucopyranose and 2.7% α-(1→3) branched chains. The Mw of this dextran was estimated to be 3.083 × 10^6^ Da and it possessed high thermal stability, WSI, WHC, and EA as well as antioxidant activity. This study not only improves the synthesis efficiency of dextran but also helps to promote research on the synthesis of LAB dextrans. Moreover, determining the properties of *Ln. pseudomesenteroides* DRP-5 glucansucrase and its biosynthetic products will aid its application in the medicine and food industries.

## Figures and Tables

**Table 1:** Emulsification activity (EA) % of dextran produced by *Ln. mesenteroides* glucansucrase with hydrocarbons and oils. (E24) 24 h; (E48) 48 h.

Hydrocarbons/Oil	EA (%)	
	*E_24_*	*E_48_*
Diesel	56.26 ± 1.79	57.50 ± 0.34
Gasoline	82.11 ± 0.52	81.67 ± 1.20
Hexane	53.82 ± 2.41	55.93 ± 1.67
Benzene	62.77 ± 0.09	60.22 ± 1.05
Soybean oil	85.53 ± 1.37	83.81 ± 0.56
Sunflower oil	70.67 ± 2.05	67.52 ± 1.72

## Data Availability

The data presented in this study are available on request from the corresponding author.
